# Deficient Grip Force Control in Schizophrenia: Behavioral and Modeling Evidence for Altered Motor Inhibition and Motor Noise

**DOI:** 10.1371/journal.pone.0111853

**Published:** 2014-11-04

**Authors:** Maxime Teremetz, Isabelle Amado, Narjes Bendjemaa, Marie-Odile Krebs, Pavel G. Lindberg, Marc A. Maier

**Affiliations:** 1 Université Paris Descartes, FR3636 CNRS, Sorbonne Paris Cité, 75006, Paris, France; 2 SHU, Université Paris Descartes, Hôpital Sainte-Anne, 75014, Paris, France; 3 INSERM U894; Université Paris Descartes, Sorbonne Paris Cité, GDR3557, Psychiatrie, 75014, Paris, France; 4 Université Paris Diderot, Sorbonne Paris Cité, 75012, Paris, France; University of Alberta, Canada

## Abstract

Whether upper limb sensorimotor control is affected in schizophrenia and how underlying pathological mechanisms may potentially intervene in these deficits is still being debated. We tested voluntary force control in schizophrenia patients and used a computational model in order to elucidate potential cerebral mechanisms underlying sensorimotor deficits in schizophrenia. A visuomotor grip force-tracking task was performed by 17 medicated and 6 non-medicated patients with schizophrenia (DSM-IV) and by 15 healthy controls. Target forces in the ramp-hold-and-release paradigm were set to 5N and to 10% maximal voluntary grip force. Force trajectory was analyzed by performance measures and Principal Component Analysis (PCA). A computational model incorporating neural control signals was used to replicate the empirically observed motor behavior and to explore underlying neural mechanisms. Grip task performance was significantly lower in medicated and non-medicated schizophrenia patients compared to controls. Three behavioral variables were significantly higher in both patient groups: tracking error (by 50%), coefficient of variation of force (by 57%) and duration of force release (up by 37%). Behavioral performance did not differ between patient groups. Computational simulation successfully replicated these findings and predicted that decreased motor inhibition, together with an increased signal-dependent motor noise, are sufficient to explain the observed motor deficits in patients. PCA also suggested altered motor inhibition as a key factor differentiating patients from control subjects: the principal component representing inhibition correlated with clinical severity. These findings show that schizophrenia affects voluntary sensorimotor control of the hand independent of medication, and suggest that reduced motor inhibition and increased signal-dependent motor noise likely reflect key pathological mechanisms of the sensorimotor deficit.

## Introduction

Since the very first description of schizophrenia, motor deficits had been noted [1], [2], in particular in gait and voluntary upper limb movements, but even today, there is no consensus on the form, the specificity and the cause of motor disturbances in schizophrenia (reviews: [3]–[6]). For skilled upper limb movements, anticipatory planning of action sequences was incriminated [7]–[9], i.e. the cognitive aspects of actions [10], [11], rather than motor control per se (but see [12]).

We investigated visuomotor grip force control, a key element of manual dexterity and a quintessential property of the sensorimotor system. It was reported that the grip/load force relation during object grasp was not affected in schizophrenia patients [7] or, if so, was in part a side effect of anti-psychotic medication [13]. Nonetheless, clinical tests and scales indicate that manual control is affected in schizophrenia [14]–[17]. Motor signs (assessed by neurological soft signs, NSS) have often been found in patients ([18]–[21]. There is little doubt that manual control is affected on a qualitative level, but it would be advantageous if quantifiable evidence of such a deficit were available.

Furthermore, it has been put forward that cortical inhibition is deficient in schizophrenia (review: [22]), including inhibition in the upper limb motor system [23]. For motor control, inhibition of muscle contraction is functionally as important as muscular excitation: it has been shown that cortical inhibition in healthy subjects, probed by transcranial magnetic stimulation (TMS) measuring short-interval intracortical inhibition (SICI), is regulated during grip force control, and varies inversely with force [24]–[25]. On the other hand it is less clear how inhibition might be affected in schizophrenia. It has been shown that patients with schizophrenia have morphological changes in cortical inhibitory interneurons mediated by γ-aminobutyric acid (GABA) [26]. Several studies have found reduced motor cortical SICI in medicated and non-medicated patients with schizophrenia [23], [27], [28], consistent with this finding.

Our goal was twofold: (i) to provide a systematic and quantitative description of a potential upper limb motor deficit in schizophrenia, (ii) to establish whether motor inhibition, probed behaviorally and computationally, is affected in schizophrenia. To this aim we used a visuomotor paradigm involving increase, maintenance and decrease of grip force, a task where modulation of motor inhibition is critical [24]. Furthermore, using a computational approach by modeling sensorimotor integration of visual, tactile and inhibitory signals for the control of force, we explored whether deficient motor inhibition might (on its own or not) explain the empirically observed deficits. We present a model based on the key assumption that only the signal gains differ between patients and controls, and no other sensorimotor mechanism.

## Materials and Methods

### Participants

Patients and control subjects were recruited from SHU-Sainte-Anne and tested in the Centre d'Evaluation et de Recherche Clinique (CERC). Three groups were included: (i) Seventeen stabilized patients (5 females, 12 males, mean age 30.0±8.4 (SD) years) that met DSM-IV-TR diagnostic criteria for schizophrenia. All were on stable atypical anti-psychotic medication for >3 months prior to the study. Concomitant medication: only stable doses of antidepressants were permitted. This group is subsequently referred to as ‘medicated patients’. (ii) Six non-medicated patients (for >6 months prior to the study) that also met DSM-IV-TR criteria for schizophrenia (2 females, 4 males, mean age 32.3±6.8 years). This group is subsequently referred to as ‘non-medicated patients’. (iii) Fifteen healthy control subjects (7 females, 8 males among the hospital staff, mean age 29.5±10.5 years). Control subjects, screened by a standardized interview, had no previous history of neurological or psychiatric illness, no familial psychiatric history and no particular dexterous skills. Demographic and clinical details for each patient and group are given in Table 1. Subjects provided informed and written consent. The study received approval by the Paris-Cochin Ethical committee and complied with the Declaration of Helsinki.

**Table 1:** Clinical and demographic details.

Medicated patients	Gender	Age (years)	Treatment duration (years)	Treatment (CE mg/day)	PANSS total	PANSS pos score	PANSS neg score	Moberg left/right (s)	MVC grip force left/right (kg)
1	M	33	4	133	63	7	22	13/14	42/45
2	F	45	10	1875	47	7	19	20/21	26/28
3	F	21	0.2	150	50	7	13	17/16	28/31
4	M	20	0.3	3750	48	8	12	18/18	41/43
5	M	39	2	500	41	7	12	13/15	44/44
6	F	26	1	470	49	9	10	18/16	29/32
7	M	40	3	400	55	7	20	18/18	46/43
8	M	37	5	200	42	7	12	17/17	55/54
9	F	24	0.2	400	83	9	26	12/12	22/24
10	M	19	0.2	300	50	7	22	15/19	39/39
11	M	44	3	583	74	17	23	20/23	50/49
12	M	30	10	330	42	7	14	18/21	40/38
13	M	33	0.5	1000	63	8	21	22/17	36/34
14	M	35	0.2	133	42	9	10	12/13	44/43
15	F	24	1	400	43	12	12	17/17	35/33
16	M	23	1	1650	45	14	11	15/15	48/57
17	M	34	4	200	71	10	21	19/15	25/25
Mean SD	12M,5F	31.0±8.4	3.2±3.4	709±867	53.4±12.8	8.9±2.9	16.5±5.4	16.7/16.9±3.0/2.9	38.2/38.9±9.6/9.6
**Non-medicated patients**									
1	M	36	na	na	53	13	12	14/16	40/48
2	F	42	na	na	67	14	13	12/12	27/27
3	M	30	na	na	45	10	9	12/12	45/49
4	M	25	na	*	47	13	9	19/15	32/39
5	F	25	na	na	68	14	13	18/17	34/29
6	M	36	na	na	63	7	15	13/13	51/52
Mean SD	4M, 2F	32.3±6.8	na	na	57.2±10.2	11.8±2.8	11.8±2.4	14.7/14.2±3.1/2.1	38.2/40.7±8.9/10.7
**Control subjects**									
Mean SD	8M,7F	29.5±10.5	na	na	na	na	na	12.9/12.6±2.0/2.0	40.1/41.7±11.2/10.9

Gender, age, duration of ongoing treatment, dose of antipsychotic treatment in chlorpromazine equivalent (CE), PANSS [32], PANSS positive score, PANSS negative score, Moberg pick up test rating (left and right hand), MVC grip force in kg (left and right hand) assessed with the Jamar hand dynamometer. Note that 40 kg corresponds to 392 N. * under antidepressants.

### Visuomotor force-tracking task

**Figure 1:**
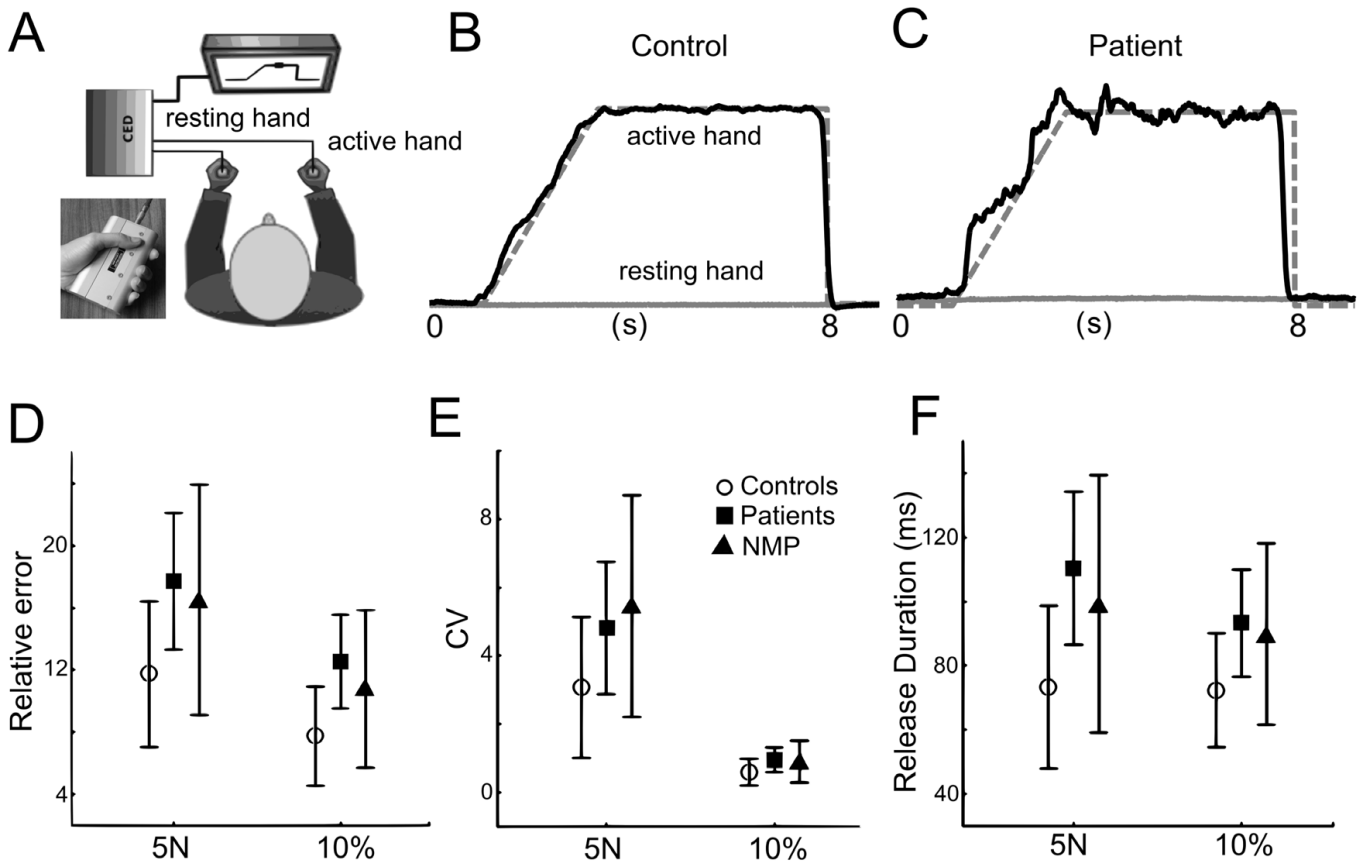
Task and behavioral results. Visuomotor grip force tracking. **A**. Setup for visual grip force tracking. The subject holds a grip force manipulandum in each hand and performs the task with the tracking hand, while holding the other manipulandum with the resting hand. Inset: Power grip manipulandum (www.sensix.com). **B**. Single-trial grip force-tracking example for a control subject at the 5N level. **C**. Corresponding example for a medicated patient. Gray stippled line: target force trajectory; black solid line: actual grip force of the tracking hand. Gray continuous line: force of the resting hand. Note larger deviation from the target in the patient compared to the control subject. **D**. Relative error (mean ±SD over the ramp and hold period) for the three groups: control subjects, medicated patients, and non-medicated patients (NMP). **E**. CV of force (mean ±SD over the ramp and hold period) for the three groups. Note that relative error and CV were higher for the low force condition (5N) since both measures are relative to target force level (c.f.^28^). **F**. Release duration (mean ±SD) for the three groups. Significant differences were found between controls and both groups of patients (see Results) in all three variables. No difference was found in force tracking variables between medicated and non-medicated patients.

A visuomotor power grip force-tracking task previously described [29] was used to assess the accuracy of force control (Fig. 1A). Grip force was recorded at 1 kHz using strain gauge force sensors linked to a CED 1401 running Spike2. The task consisted of a series of visually displayed ramp-hold-and-release target force trajectories to be followed as closely as possible with a cursor (moving vertically as a linear function of grip force), while the target force trajectory scrolled continuously over the screen from right to left. Upcoming force was thus predictable. For both low and high force conditions, the pre-ramp (inter-trial) period lasted 3 s, the ramp period 2 s, and the hold period 4 s, after which the target force dropped instantaneously to baseline (0N). Force-tracking was performed once with the right and once with the left hand (pseudo-randomized across subjects). While tracking with one hand, the other (resting) hand remained passive but still gripped a manipulandum, so that unwanted motor overflow could be quantified. Each task condition consisted of 16 trials. Condition_1: low absolute force level (5N). Condition_2: higher relative force level (10% maximal voluntary grip force, MVC). Trials were grouped by force level in blocks of four trials, and four blocks were performed at each force level (total of 32 trials). Subjects were instructed to minimize the distance (error) between the applied and the target force and to release force immediately at the end of the hold phase. All subjects were familiarized with the task before testing.

The following single-trial performance measures were computed [29] based on the 100 Hz down-sampled force signal:

- **Relative error (dimensionless).** Total error over time (area) between the applied force and the target force trajectory normalized to the target force level. Note: an identical total error at two different force levels will lead, due to normalization, to a smaller relative error at the higher level (c.f. [30]).
- **Variability.** Coefficient of variation (CV) of force (i.e., SD/mean). The CV expresses variability relative to the mean force level.
- **Release duration (ms).** Time taken to abruptly reduce the grip force from 75% to 25% of the target force.
- **Release onset (ms).** Time of initial force reduction, quantified as the time when the slope (dF/dt) first crossed a negative threshold, i.e., dF/dt<-6N/s.
- **Force onset (ms).** The time when the slope (dF/dt) of the applied force crossed a positive threshold, i.e., dF/dt>0.2*max (dF/dt). Expressed with respect to target ramp onset.
- **Motor overflow (N).** Mean force of the resting hand for three separate 1 s periods (during baseline, ramp and hold) relative to those of the tracking hand.

### Data analysis and statistics

Force data were analyzed using Matlab v7 (The MathWorks, Inc., Natick, MA, USA). Statistical analysis was performed using Statistica 10 (StatSoft, Inc., Tulsa, OK, USA). Relative error was analyzed using a general linear model repeated measures ANOVA with one GROUP factor (medicated/non-medicated/controls) and three within-subject factors: PHASE (ramp/hold), HAND (dominant/non-dominant) and FORCE (5N/10%). CV, release duration, and release onset were analyzed in the same way. Post-hoc Fisher LSD tests were applied for significant differences. The level of significance was set to p≤0.05.

**Figure 2:**
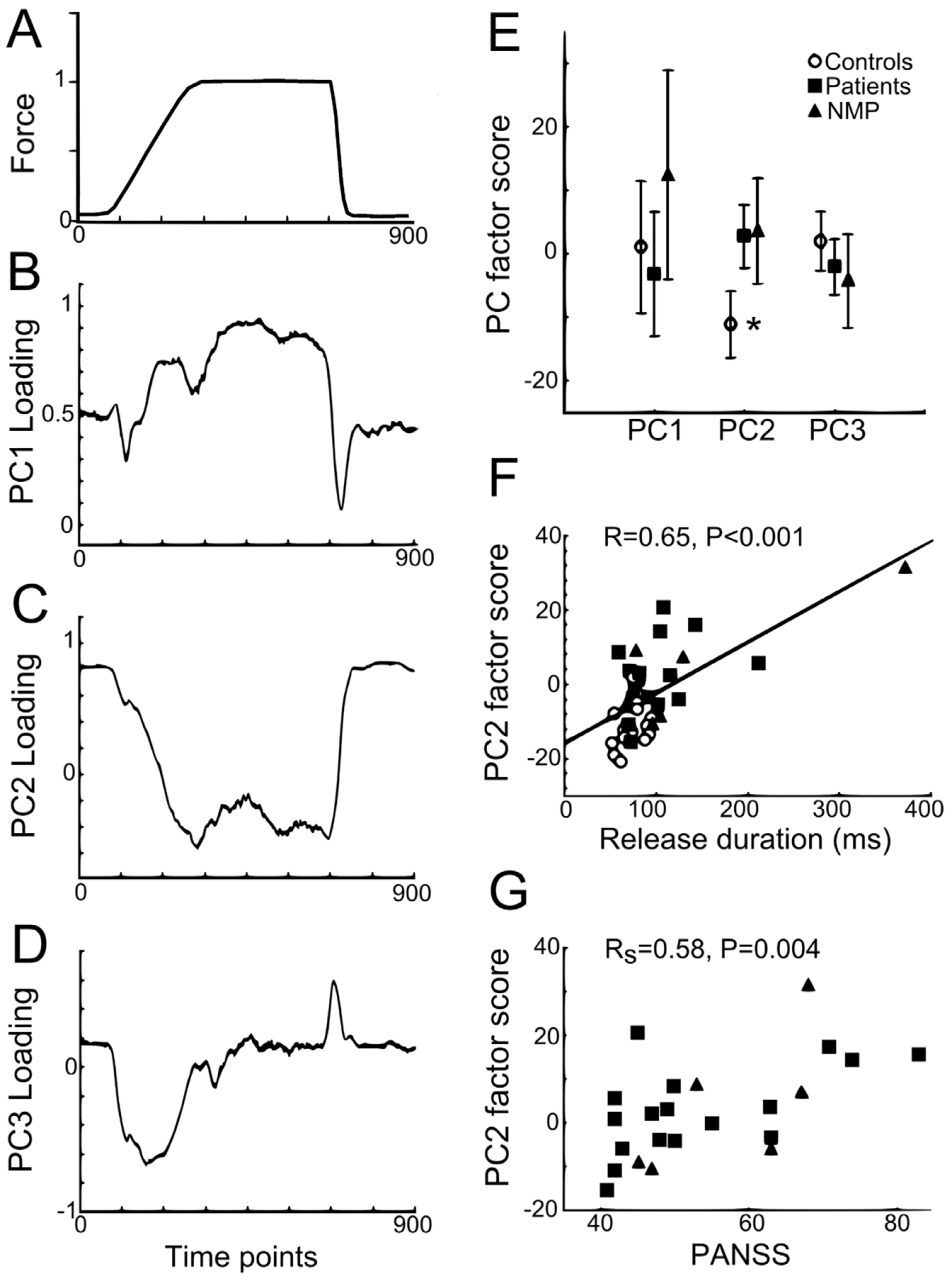
PCA of force tracking traces across subjects. **A**. Average force trace over all conditions and subjects (N = 38). **B–D**. PC loading as a function of time for PC1, PC2 and PC3, respectively. **B**. Loading profile similar to force profile for PC1, positive and increasing scores during ramp, more stable and strongest positive scores during hold. **C**. Inverse loading profile compared to force for PC2. **D**. Strongest loading during force transitions (ramp and release) for PC3. **E**. Average factor score (±SD) for PC1, PC2 and PC3 for control subjects vs medicated patients, and non-medicated patients (NMP). Significant difference between controls and both groups of patients only found for PC2 (more negative scores for controls: asterisk). **F**. Positive correlation between PC2 factor score and release duration for control subjects and patients. Correlation remained significant with exclusion of outlier subject (p = 0.003). No correlation was found between PC2 factor score and relative error or CV (p>0.5). **G**. Positive rank correlation between PC2 factor scores and PANSS scores in patients.

**Figure 3:**
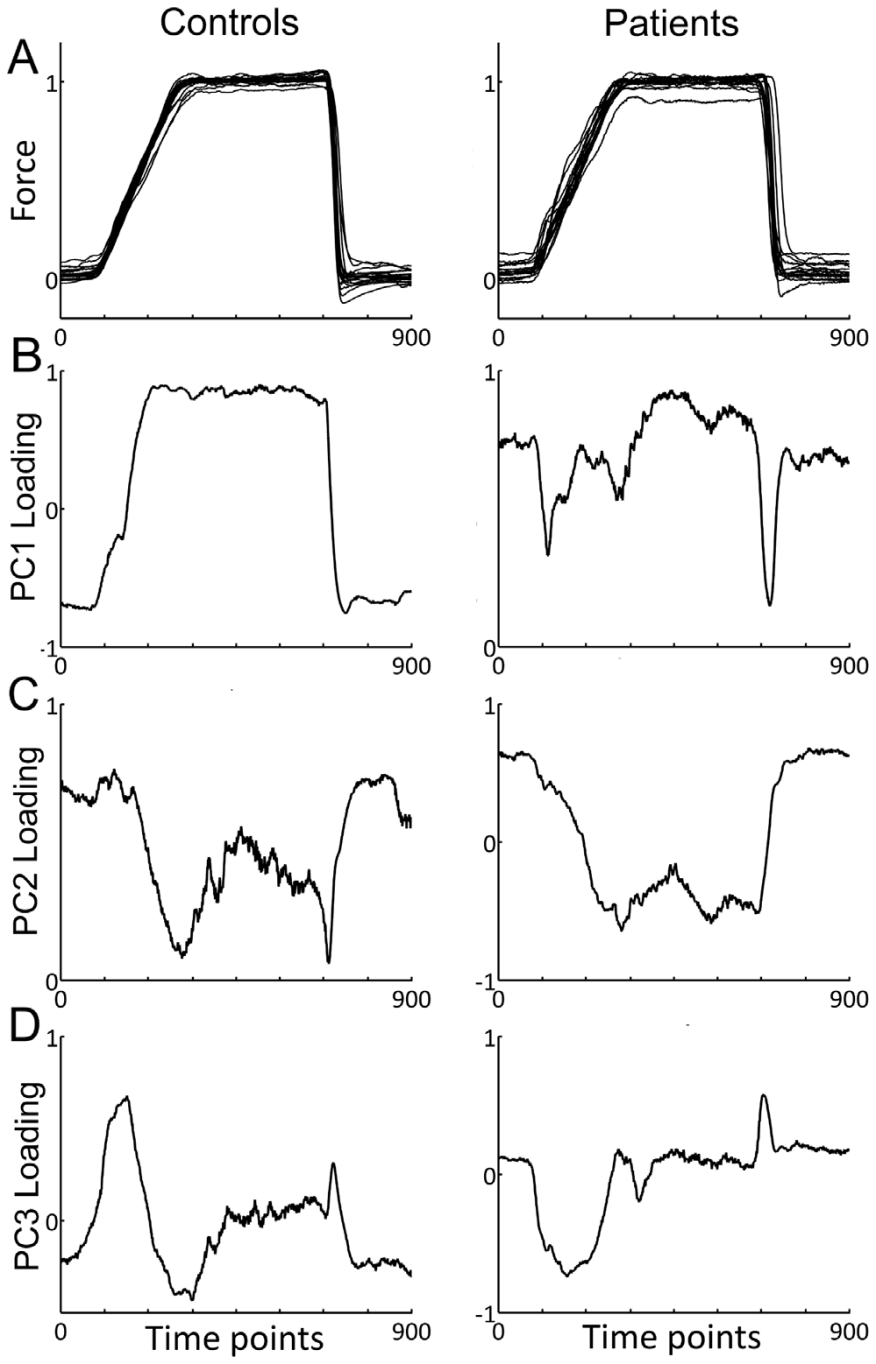
Separate PCA of force tracking traces for control subjects and patients. **A**. Average force trace for each control subject (left) and each patient (medicated and non-medicated patients pooled, right). Note higher variations of baseline force in patients. **B–D**. PC loading as a function of time for PC1, PC2 and PC3, respectively. **B**. PC1 loading as a function of time for controls (left) and patients (right). Strong resemblance to force trace present in controls, less so in patients. **C**. PC2 loading as a function of time for controls (left) and patients (right). Resemblance to the inverse force profile in both groups. **D**. PC3 loading as a function of time for controls (left) and patients (right). Strongest loading during force transitions (ramp and release) for both groups.

Principal Component Analysis (PCA) may identify underlying control strategies, e.g., for human grasp kinematics [31]. We performed a PCA on the mean force with the aim to split the time-varying force profile into PCs and to check whether these were different for the three groups. This PCA was performed across all subjects in order to compare groups with respect to their common PCs (Fig. 2). We used the nonlinear iterative partial least squares (NIPALS) method on the average force trace for each subject. The PCA data consisted of a 900×38 matrix (900 force samples representing 9 s of the trial- and condition-averaged force trace, times 38 subjects). PC factor scores were compared in an ANOVA with one GROUP factor. In addition, a separate PCA for the control subjects (900×15) and for the patients (900×23) was performed for a more qualitative comparison between groups (Fig. 3). We use the term ‘qualitative’ since there is no mathematical guarantee that the resulting PCs in each group are co-linear and ordered identically (in terms of explained variance) due to the difference in the underlying covariance matrix of each group.

Relations between force tracking variables were investigated using Pearson's correlation, and relations between force tracking variables and symptom severity (PANSS score [32]) were investigated using Spearman rank correlations (R_S_). A Bonferroni correction for multiple comparisons was applied.

### Computational model of sensorimotor integration

**Figure 4:**
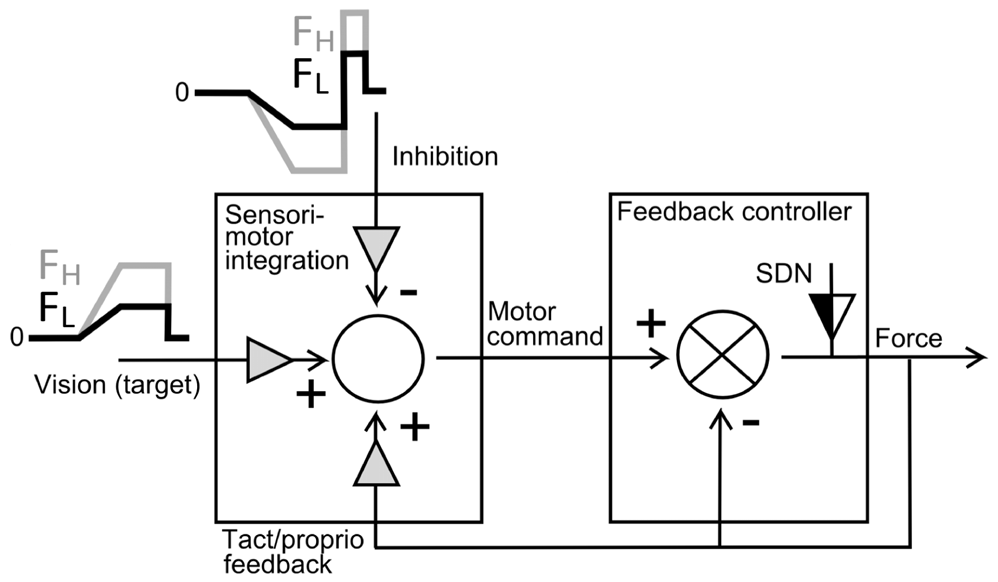
Block-scheme of the computational model of sensorimotor integration for grip force tracking. Three input signals are integrated to form a motor command: (i) visual information on the ramp-hold-and-release target force trajectory for two different force levels (F_L_, F_H_ for low and high force, respectively), (ii) inhibition modulated as a function of target force (stronger modulation for high force F_H_, weaker modulation for low force F_L_ condition), and (iii) tactile/proprioceptive feedback (a function of force). Each input signal has a gain (gray triangle): the sum of these gains needs to be  = 1. Grip force (the model output) is regulated by a negative feedback-controller as a function of the error between the motor command and the actual grip force. Signal-dependent noise (SDN), with an adjustable gain (black-and-white triangle) is added to the grip force. The goal is to simulate empirically observed behavioral differences between patients and control subjects. Main assumption: a change in gains is sufficient to explain the behavioral difference.

A simple computational model of sensorimotor integration was implemented under Simulink (The MathWorks, Inc., Natick, MA, USA). The goal was to replicate empirical (behavioral) differences between the patient and the control group and to explain these differences in terms of the underlying neural control signals used in the model. This model (Fig. 4) considered three types of time-varying input signals to be integrated and to form a motor command: (i) visual information on the ramp-hold-and-release target force trajectory, (ii) inhibition modulated as a function of target force, and (iii) tactile/proprioceptive feedback. Each input signal had its own gain: V_Gain, I_Gain and TP_Gain, respectively. This motor command then entered a negative feedback-loop where the grip force (the model output) was dynamically regulated as a function of the error between the motor command and the actual grip force. Signal-dependent noise was added to the grip force. The following assumptions have been implemented:

### Neural input signals

- Visual input: a time-varying form according to the empirically used target force trajectory.
- Inhibitory input: a time-varying form shown in Fig. 4 and consistent with empirical observations [24], [25]. Inhibition decreases with increasing force [24], remains constant during hold, and there is a phasic increases at the moment of force release [25]. White noise was added to the visual and inhibitory signals.
- Tactile/proprioceptive input: a feedback (downscaled copy) of the time-varying grip force, consistent with tactile and proprioceptive afferents [33], as well as with cells in somatosensory cortex [34], that have been shown to increase their firing with force.
- Gains on input signals: visual gain (V_Gain), inhibitory gain (I_Gain) and gain of tactile/proprioceptive input (TP_Gain). These add up to unity.

### Sensorimotor integration

- Motor command: the sum of the visual, inhibitory and tactile/proprioceptive input. For the motor command to scale as a function of the target force, the sum of the gains of the three input signals needs to add up to unity.

### Output signal

- Grip force: is controlled by a negative feedback loop that integrates the error, i.e. the difference between actual grip force and the motor command. Linearly increasing signal-dependent noise (SDN) was added to the grip force (in line with [35]).

### Simulation

- General assumption: patients differ from control subjects in terms of altered gains, i.e. there is no alteration in the mechanism of sensorimotor integration or in the temporal characteristics of the input signals. If so, altered gains should be sufficient to simulate the empirically observed behavioral differences between patients and controls.
- Simulation procedure: twenty runs (each trial with different, pseudo-randomized seeds for the noise) were computed for each condition. Performance measures were calculated per trial and averaged across trials for a given condition (as for the empirical data).
- Assumptions on gains: V_Gain was arbitrarily set to 0.5 and constant for all conditions. For control subjects I_Gain was set to 0.2, corresponding to 20% of inhibitory cortical cells and synapses [36], and therefore TP_Gain was 0.3.
- Tunable parameters: SDN_Gain for control subjects. SDN_Gain and I_Gain (TP_Gain) for patients. All other parameters remained constant for all conditions.

## Results

### Clinical and functional assessment

Medicated patients, non-medicated patients and controls did not differ in age, gender, tactile sensitivity (Semmes-Weinstein test) or MVC grip strength (Table 1). Medicated patients had greater negative PANSS score (p = 0.004) than non-medicated patients, but did not differ in the other scores (Table 1). Both medicated and non-medicated patients took longer time to complete the functional dexterity (Moberg pick-up) task, but only medicated patients differed significantly (p<0.01) (Table 1).

### Sensorimotor force tracking performance

All subjects completed the tracking task successfully (Fig. 1B, C), in the 5N condition (low target force), as well as in the 10% MVC condition (high target force, i.e. 38±9N in patients, 40±10N in controls). Target force in the 10% MVC condition was therefore about 8 times higher than in the 5N condition. Compared to healthy control subjects, medicated and non-medicated patients showed a decreased accuracy of visuomotor grip force control. All three performance measures were affected: relative error during ramp and hold (Fig. 1D), CV of force (Fig. 1E), and release duration (Fig. 1F). The ANOVA showed a significant effect of GROUP for relative error (F(2,35) = 10.7, p<0.001, η^2^_p_ = 0.379), with both groups of patients producing ∼50% more error than controls. Post-hoc comparisons showed differences between medicated patients and controls (p<0.001) and between non-medicated patients and controls (p = 0.02). Release duration also differed significantly according to GROUP (F(2,35) = 4.7, p = 0.01, η^2^_p_ = 0.21) and was ∼35% longer in patients. Post-hoc comparisons showed differences between controls and medicated patients (p = 0.005), but not between non-medicated patients and controls (p = 0.11). Force variability (CV) was also increased in both groups of patients (GROUP: F(2,35) = 4.7, p = 0.01, η^2^_p_ = 0.21), by ∼50%. Post-hoc comparisons showed differences between controls and medicated (p = 0.01) and non-medicated (p = 0.01) patients. There was no difference between medicated and non-medicated patients in error, release duration or CV (post-hoc error: p = 0.35; release duration: p = 0.58; CV: p = 0.58).

Error and release duration were similar in dominant and non-dominant hands, i.e. no effect of HAND (p>0.05), whereas, CV was higher in the dominant hand (F(1,35) = 11.5, p = 0.002, η^2^_p_ = 0.25). Posthoc tests showed that this difference in CV between hands was present in the three groups, and only at the 5N level (p<0.05).

ANOVA showed a significant FORCE effect on relative error (F(1,35) = 52.1; p<0.001; η^2^_p_ = 0.60) and CV (F(1,35) = 141.6; p<0.001; η^2^_p_ = 0.80), and a smaller effect on release duration (F(1,35) = 4.6; p = 0.04; η^2^_p_ = 0.12). For all three groups post-hoc comparisons revealed higher relative error (Fig. 1D) and a higher CV (Fig. 1E) at 5N compared to 10% MVC. No interaction was found between FORCE*GROUP. Mean error (across hands) correlated with mean release duration (r = 0.50, p = 0.001) and mean CV (r = 0.51, p = 0.003). Timing of force onsets and offsets were similar in all three groups (p>0.2).

Motor overflow to the resting hand was not visibly increased in patients (Fig. 1B, C) and no GROUP difference was found (F(2,35) = 0.6; p = 0.58; η^2^_p_ = 0.03).

### Principal Component Analysis of force tracking across subjects

The PCA identified 19 PCs. The first three PCs explained a total of 83% of the variation in the force trace (PC1 explained 44%, PC2 31%, PC3 8%). The remaining 16 PCs explained less than 5% each. Fig. 2A shows the grand average force trace across all subjects and Fig. 2B–D show the corresponding *loading* scores for the first three PCs. The loading represents the information shared by (correlation between) a given PC and the time-varying force data. Qualitatively, the time-varying PC1 loading resembled the target force trajectory (Fig. 2B), the envelope of the PC2 loading resembled the inverse of the force trajectory (Fig. 2C), whereas the shape of the PC3 loading resembled the inverse of the first derivative of force (Fig. 2D). The *factor* scores represent the distance of each subject's data to the origin of each PC: a significant GROUP effect was found for PC2 scores (F(2,35) = 9.1; p<0.001; η^2^_p_ = 0.34), with PC2 factor scores being lower in controls compared to both medicated (p = 0.006) and non-medicated patients (p = 0.03) (asterisk in Fig. 2E). No group difference was found in PC1 or PC3 factor scores.

### Separate principal component analysis of force tracking for controls and patients

For a more qualitative comparison we also performed a separate PCA for the two groups. For control subjects (N = 15) the first three PCs explained a total variance of 85% (PC1 explained 55%, PC2 24%, PC3 6%). Similarly for the patients (N = 23), the first three PCs explained a total variance of 84% (PC1 explained 52%, PC2 23%, PC3 9%). Figure 3A shows the average force trace for each subject, for controls (left) and for patients (right). Except for somewhat more variability during the baseline, there was no obvious difference between controls and patients. However, the loading profiles of the first three PCs showed qualitative differences (Fig. 3B–D). The PC1 loading profile of the controls was comparable to the force trace, which was not obvious for the patients. Nonetheless, the PC1 loading across all subjects (Fig. 2B) represented (on first approximation) the average of the two separate PC1 loadings. The PC2 loadings were qualitatively similar between the two groups (Fig. 3C) and similar to the profile across all subjects (Fig. 2C). The PC3 loadings were highest during the force transitions, for controls as well as for patients (Fig. 3D), as was the case for the PCA across subjects.

### Relation between force tracking, PCA and clinical scales

For the PCA across subjects, a positive correlation was found between PC2 factor score and release duration across all subjects (Fig. 2F), but not for PC1 or PC3. Relative error also correlated positively with PC2 (R = 0.58, p<0.001) and negatively with PC3 (R = −0.66, p<0.001). Furthermore, in patients a positive correlation was observed between PC2 factor scores and the total PANSS scores (Fig. 2G) and with positive, negative and general sub-scores. No other significant correlations were obtained between clinical scales and force tracking data.

### Model predictions

#### Simulation procedure

We modeled low-force trials and 8-times larger high-force trials, corresponding to the empirical difference between the 5N and 10% MVC condition. Three performance measures were calculated similar to the empirical data: relative error as well as CV during the hold period, and release duration.

#### Fitting procedure

**Figure 5:**
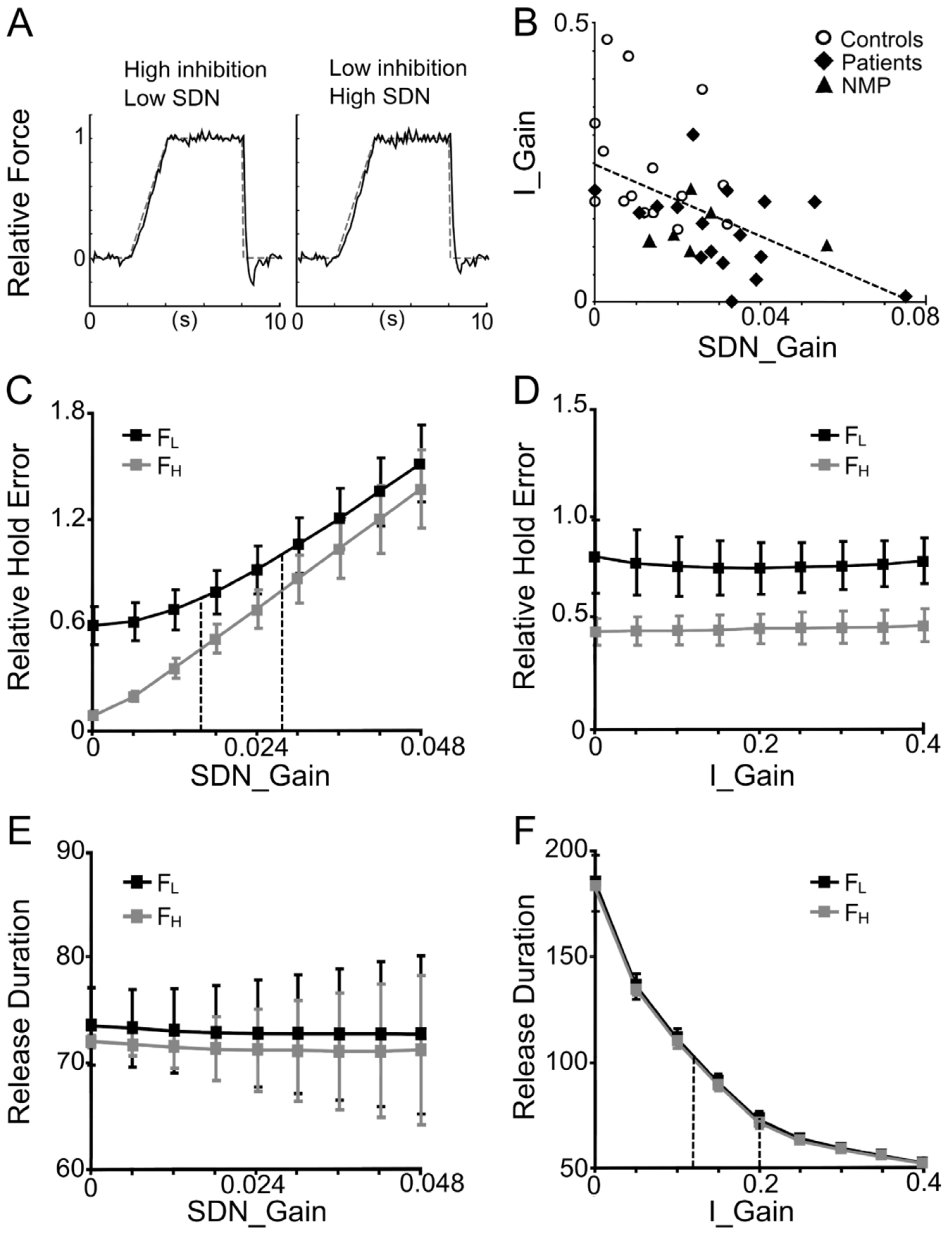
Model data: functional consequences of gain changes. **A**. Single trial runs with identical seed for a simulated average control subject (left) and an average schizophrenia patient (right) at the low force level. **C–F**. Performance measures as a function of gains. Twenty runs with pseudo-randomized initial seeds were computed for each condition. Performance measures (mean ± SD) were calculated similar to the empirical data. Black: low force condition (F_L_), gray: high force condition (F_H_). **C, E**. Influence of SDN-gain on relative error (C) and on release duration (E). Increasing SDN-gains provides higher relative error (and higher CV, not shown), but has no effect on release duration. In C, stippled vertical lines indicate the average SDN_gain for controls (0.016) and patients (0.028). **D, F**. Impact of inhibition-gain on relative error (D), and on release duration (F). Increasing inhibitory gain has little effect on relative error (and CV, not shown), but decreases the release duration. In F, stippled vertical lines indicate the average I_Gain for controls (0.2) and patients (0.12). Note that, for a given gain, error and CV are always higher for the low force compared to the high force condition (c.f. Fig. 1C, D). **B**. Relation between I_Gain and SDN_Gain after fitting the gains to each subject's performance. There is a significant negative correlation (regression line stippled), across the whole population [controls, medicated patients, and non-medicated patients (NMP)], with patients tending to have lower I_Gains and higher SDN_Gains. Note: this resembles the correlation found empirically between mean error and release duration (see Results).

At the group level (Table 2), SDN_Gain was first set such that the ratio of relative error (and of CV) between low and high force conditions was as close as possible to the empirical observed ratios. This was done separately for the grand average of controls and of patients. Second, I_Gain (and therefore TP_Gain) was set such that release duration was as close as possible to empirical values, again done separately for the grand average of controls and of patients (Table 2). In addition, for comparison at the individual level (Fig. 5B) this fitting procedure was applied subject-by-subject.

**Table 2:** Comparison between model and empirical performance in grip force tracking.

		Model data		Empirical data	
		Low force	High force	Low force (5N)	High force 10% MVC
Relative hold error	Controls	0.75 (ref)	62%	9.8 (ref)	61%
	SCZ	134%	106%	145%	108%
CV hold	Controls	0.03 (ref)	62%	0.028 (ref)	57%
	SCZ	133%	106%	129%	89%
Release duration	Controls	73 ms (ref)	98%	73 ms (ref)	99%
	SCZ	140%	138%	143%	127%

Relative error during the hold period (Relative hold error), Coefficient of variation of force during the hold period (CV hold), release duration for controls and for patients (SCZ). For each variable, the ‘low force, control subject' data was taken as reference (ref) and the comparative values of the three other conditions expressed as %reference. Note that the large difference between the relative hold error (the reference values of the controls) between the model and empirical data is arbitrary, since the modeled force is dimensionless. This is the reason for the percentage-based comparison.

#### Results

Figure 5A shows simulated single-trial force-tracking for controls (left) and for patients (right). Optimal group gain-settings for controls were: SDN_Gain  = 0.016, I_Gain  = 0.2, TP_Gain  = 0.3. With these settings relative error and CV decreased in the high force condition, whereas release duration remained unchanged, as was shown empirically (Table 2, c.f. Fig. 1D–F). For patients optimal group settings were: SDN_Gain  = 0.028, I_Gain  = 0.12, TP_Gain  = 0.38. With these conjoint settings (larger SDN_gain, smaller I_Gain) the model performance showed increased relative error, CV and release duration in both low and high force conditions compared to controls, consistent with our empirical data (Table 2, c.f. Fig. 1D–F). These gain differences between controls and patients are also illustrated in Fig. 5C, F (stippled lines).

Systematic variation of these two critical gains showed that increased SDN in the motor output produces more error (and higher CV, not shown), but does not affect release duration (Fig. 5C, D). In contrast, reduced inhibition generates longer release duration, but has little or no effect on tracking error (Fig. 5D, F) or CV (not shown).

Fitting I_Gain and SDN_Gain to each subject's performance and plotting the gain space revealed that I_Gain was significantly and negatively correlated to SDN_Gain (Fig. 5B). This held across all subjects (r = −0.53, p<0.001), as well as within the patient group (r = −0.42, p<0.05). Again, patients tended to have lower I_Gains and higher SDN_Gains, in spite of substantial overlap. Furthermore, across subjects I_Gain also correlated significantly (r = −0.54, p<0.001) with the PC2 factor scores of the PCA.

## Discussion

We found that schizophrenia patients (medicated and non-medicated) had significant deficits in visuomotor grip force-tracking: patients showed less accuracy in force modulation (higher tracking error), increased force variability (higher CV) and prolonged stopping of grip force (longer release duration). Nonetheless, all subjects achieved task completion. These significant motor deficits were also present in non-medicated patients, although sample size was small. This suggests that atypical antipsychotics cannot account for the observed alterations. This finding, however, needs confirmation in a larger sample (note on feasibility: pharmacological treatment was initiated without delay when clinically indicated). The relatively low PANNS scores of the non-medicated patients indicate that they had not yet relapsed and were thus comparable to medicated patients.

### Model predictions on neural mechanisms

The potential mechanisms underlying the three observed tracking deficits have been investigated by a computational model which successfully replicated the behavioral characteristics of control subjects and of schizophrenia patients. The model suggests that reduced motor inhibition as well as increased SDN in the motor output account for these deficits: neither inhibition on its own, nor SDN explained the three empirically observed deficits (error, CV, release duration), but the combination of the two did. These two clear-cut model-predictions require empirical verification. Furthermore, I_Gain and SDN_Gain were correlated: this suggests that decreased motor inhibition may cause increased SDN. These results need to be understood within the four key-assumptions (constraints) of the model:

- *Inhibition* was not constant, but varied as a function of force. Its time-varying form is consistent with empirical data: it was shown that short-latency intracortical inhibition (SICI) decreases with increasing grip force [24] and increases with muscle relaxation [25]. This increase with relaxation was modeled as a phasic inhibition during release: in the absence of this signal, variations of I_Gain did no longer affect release duration. Furthermore, this inhibitory profile is akin to decreased firing with increasing grip force found in subpopulations of primary motor (M1) and sensorimotor (S1) neurons in the non-human primate [34], [37]. In schizophrenia, several studies have found reduced SICI in medicated and non-medicated patients [23], [27], [28], in accordance with our modeling results.
- *SDN* was added to the motor output (force) and white noise to visual and inhibitory signals, consistent with noise being present in all stages of sensorimotor control [38], including sensory [39] and signal-dependent motor noise [40]. Reduced signal-to-noise ratio in cortical processing has been highlighted in previous computational models of schizophrenia [41], [42] and is in line with less focused cortical processing observed in schizophrenia [43]–[45].
- *Multimodal* sensory (visual and tactile/propriocptive) signals were summed to form the motor command. The interaction of tactile/proprioceptive [33] and visual cues [46] for grasp and force control has been extensively studied. Although the mathematical form of the integration remains unknown, there is no doubt that multi-modal interaction occurs at the cortical level (reviews: [47], [48]).
- *Signal gains*: we assumed that patients with schizophrenia differed in gains, but not in other terms. This seems justified since patients with schizophrenia had no problems with task completion and no deficits in tactile or visual perception. Adding different time-varying signals for different groups would have lead to self-fulfilling results. A lower inhibitory gain in patients is compatible with patients being different from controls in terms of weaker modulation, but not in terms of absence of motor inhibition. Many studies showed an inhibition deficit in oculomotor [49] and manual tasks [50]: in particular, Badcock *et al*. [51] concluded, in line with our results, that patients had a deficit in modulating inhibition during movement execution.

### PCA and motor inhibition

If PCs extracted from behavioral data reflect underlying control strategies (e.g. [31]), then our PCA across subjects would suggest altered neural control strategies in schizophrenia patients. The time-varying loading scores of each PC represented a distinct envelope: resembling the target force trajectory (PC1), its inverse (PC2) and its inverted derivative (PC3). Interestingly, PC loading profiles resembled cortical single cell activity: many M1 cells ([34], [37]), qualitatively similar to PC1 loading, increased their firing during ramp-and-hold precision grip force control, whereas some cells were activated with changes in force (as PC3), and some decreased firing as force increased (as PC2).

Remarkably, the PC2 scores, which we interpreted as an inhibition, were significantly different in patients compared to control subjects, but not between patient groups. These PCA results are thus entirely coherent with the predictions from the computational model: both suggest a task-related deficiency (weaker modulation) of motor inhibition in schizophrenia similar to that found in the oculomotor system [49], [58].

Separate PCA for the control group and for patients showed qualitative differences in the time-varying loading scores: this was obvious for PC1, whereas the loadings for PC2, which potentially reflect the time-course of inhibition, showed only marginal differences. This suggests that not the time-varying profile, but the gain (reflected by the PC factor score) varies among control subjects and patients, consistent with the model-prediction.

Furthermore, PC2 factor scores correlated positively with the empirically observed release duration, as well as with the PANSS symptom score. This suggests that the PC2 component may be a marker of reduced inhibition and may be indicative of more generalized pathological mechanisms, in line with other studies indicating that inhibition is linked to disease severity [59], [60].

### Interpretational limits of the model

The interpretational limits of the computational model depend on both the incorporated and the non-incorporated constraints. The rational of the model was not to provide a novel theory of motor control but to quantify the motor consequences of gain changes in the interaction of sensory and motor signals. The model is compatible with most control theories, since its basic element is the feedback loop [52]. We will examine three limits: the exclusion of other explaining factors, neural topology and clinical specificity. (i) The model does not exclude other factors that may account for schizophrenia-specific deficits. In particular, the substantial overlap in gain values between schizophrenia patients and controls (Fig. 5B) suggests that additional factors intervene in schizophrenia-specific sensorimotor integration deficits. Nonetheless, the model-based result that reduced inhibition and increased SDN both produce motor deficits are two clear-cut predictions, which require further empirical verification. Moreover, the model also shows that these two factors are not completely independent suggesting that decreased inhibition may cause increased SDN. (ii) Since the model did not include any topological (structural) constraints, it does not strictu senso predict that the motor cortex is the neural structure which produces the modeled (and behaviorally observed) deficits. Therefore, an implication of the basal ganglia in force control [53] and/or the cerebellum [54] cannot be excluded. Nonetheless, we think that altered inhibition in the cortical motor areas is likely the cause of the observed motor deficit, as M1 is not only the main locus for motor execution, but also for stopping a motor action [55]. However, this needs further empirical verification. In accordance with our assumption, terminating a motor task has been shown to rely on inhibition within M1 [56], [25], and reduced inhibition within M1 correlates with increased disease severity in schizophrenia [28]. (iii) Whether the observed motor deficits and their computational explanation are specific to schizophrenia remains debatable. The computational results, including deficient cortical inhibition and altered motor noise as potential mechanisms, do not imply any specificity. Whether quantitatively similar behavioral deficits occur in other patient groups (e.g. Parkinson disease [57]) needs further investigation.

## Conclusions

There is no reason to suppose that the sensorimotor system is exempt from the hypothesized neuro-developmental disorder underlying schizophrenia [61]. Consistent with this, our study shows clear, quantitative visuomotor grip force control deficits, independent of medication. The three affected performance measures may form a behavioral marker of schizophrenia. Our computational model as well as the PCA suggest that reduced modulation of inhibition and increased signal-dependent motor noise likely reflect the neuropathological mechanisms of the observed sensorimotor deficit in schizophrenia.
